# Topical Minoxidil Overdose in a Young Man With Androgenetic Alopecia: A Case Report

**DOI:** 10.7759/cureus.62382

**Published:** 2024-06-14

**Authors:** Maria A Ponomareva, Maria A Romanova, Anastasia A Shaposhnikova, Gennadii A Piavchenko

**Affiliations:** 1 Human Anatomy and Histology, I.M. Sechenov First Moscow State Medical University, Moscow, RUS; 2 Clinical Medicine, I.M. Sechenov First Moscow State Medical University, Moscow, RUS

**Keywords:** 2% topical minoxidil, rare side effect, androgenetic alopecia, drug-induced hypotension, cardiovascular side effects, minoxidil intoxication

## Abstract

Minoxidil is an effective and relatively safe topical drug that is used to treat androgenetic alopecia and other types of alopecia. This active ingredient is used in dermatology as a hair growth stimulant; however, the use of solutions containing minoxidil can be accompanied by a variety of cardiovascular systemic side effects. In this case report, we describe the case of a 23-year-old man who presented with complaints of dizziness, blurred vision, general malaise, fatigue, and feeling pre-syncopal while standing after applying large amounts of topical minoxidil solution for three days in a row. Other potential causes of the presenting condition were excluded. The symptoms quickly resolved after the discontinuation of minoxidil. No other treatment was used apart from minoxidil withdrawal.

## Introduction

Minoxidil is an FDA-approved component of over-the-counter solutions used to treat androgenetic and other types of alopecia [1]. This active ingredient is used in dermatology owing to its ability to trigger hypertrichosis; however, the use of formulas containing minoxidil can be accompanied by a variety of systemic side effects in rare cases [2-4]. We report a case of systemic cardiovascular effects of topically administered excessive amounts of minoxidil in a young man.

## Case presentation

**Figure 1:**
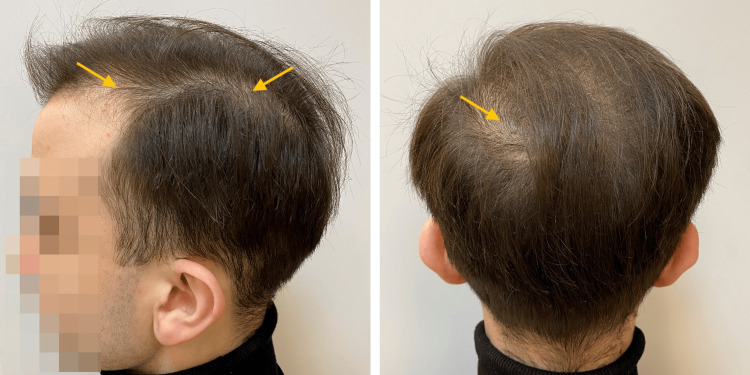
The patient - a young man with androgenetic alopecia. Appearance at the time of first clinical admission. The signs of diffuse hair loss (yellow arrows).

A 23-year-old white man presented with complaints of intensive dizziness for three days (Figure 1). Other symptoms included blurred vision, general malaise, and fatigue. He also complained of worsening of vertigo and fatigue while standing, and feeling pre-syncopal. The patient denied taking any medication or prohibited substances, and his medical history was of no significance. He was vaccinated according to the immunization schedule in Russia, was not predisposed to seasonal colds, and did not undergo any operations. He had not left the country for a year before admission. He had no history of getting psychiatric treatment and showed no signs of emotional, behavioral, or cognitive disorders, overall appearing healthy.

According to the information provided by the patient, he had started using 2% minoxidil topical solution regularly for his manifesting androgenetic alopecia six months before the onset of symptoms and was satisfied by the results of minoxidil treatment. After the spray cap of the bottle with the solution he was using had been broken, he could not properly regulate the amount of product applied to his scalp. As told by the patient, he had been applying topical minoxidil in amounts of about half of a teaspoon twice a day for the last few days before the admission and was suffering from the symptoms described above.

The decision was made to carry out a differential diagnosis according to the European Society of Cardiology guidelines on syncope (2018) in order to exclude cardiac pathology. The clinical examination of the patient during the first admission was unremarkable, excluding his average resting blood pressure of 90/58 mmHg (it was measured three times during the appointment, resulting in 89/58, 91/59, and 91/58 mmHg, accordingly). His heart rate was 84 bpm on average after triple measurement (87, 82, 83 bpm accordingly). Cardiac auscultation revealed a regular rhythm without a murmur. An ECG revealed a regular sinus rhythm; the values of his complete blood count and blood glucose were within the reference values. A dermatological examination of the scalp did not bring new data to the clinical picture as the skin appeared completely normal, without any signs of inflammation, rash, discoloration, or findings of an infectious or traumatic nature.

The patient was recommended to discontinue minoxidil use, and his symptoms disappeared without any special treatment the day after minoxidil withdrawal. Apart from discontinuation of minoxidil, no other treatment was used. At a follow-up appointment a week after the first one, the patient’s resting blood pressure was 110/75 mmHg.

## Discussion

Minoxidil was first introduced in the 1970s as an antihypertensive drug, and the discovery of its common side effect, hypertrichosis, led to the development of a topical formulation to stimulate hair growth [5]. Common side effects of topical minoxidil use include several dermatological reactions of allergic and non-allergic nature, such as contact dermatitis and seborrheic keratosis [6]. Although minoxidil has a reputation for being a safe component for treating androgenic alopecia, the use of minoxidil-containing products can be accompanied not only by local dermatological reactions but also by a variety of systemic effects, such as hypotension, syncope, paresthesia, or chorioretinopathy [2-4,7]. The number of case reports of topically applied minoxidil intoxication or systemic side effects is much smaller than the number of reports of minoxidil poisoning after oral intake. At the moment, the possibility of using tablet forms of minoxidil to treat various types of alopecia in men and women is being studied [8-10]. Nevertheless, minoxidil causes severe toxicity when sufficiently ingested [11-13].

The mechanism of hair growth intensification by minoxidil is not fully understood although it is believed to prolong the anagen phase in hair follicles [1,5]. There is evidence that minoxidil may act by altering hormonal and enzymatic pathways, which results in lowering the ability of hair follicles to bind dihydrotestosterone and enhancing the production of estradiol [14].

When applied externally, the systemic uptake of topical minoxidil is approximately 1.4% of the amount applied to the scalp; however, this value may vary depending on the condition of the skin, the application frequency, and the composition of this substance in the products used [5].

The development of hypotension and syncope as a systemic side effect of topical minoxidil in a young man was described by Dubrey et al. (2015) [2]. Any other potential causes of the presented condition were excluded, and after the discontinuation of minoxidil, the symptoms quickly resolved. According to a study described by Ranchoff R. E. and Bergfeld W. F. (1985), seven of 30 patients treated with a topical solution with a concentration of minoxidil of 3% twice a day developed asymptomatic arterial hypotension, whereas no increase in heart rate was recorded [7].

In this particular case, the patient was examined for cardiac pathology in the first place, because potential arrhythmogenic causes of the described symptoms (and presyncopе being the most alarming of them) were rated as prognostically worst. The relationship between the developed condition and the use of topical minoxidil seems obvious, given the evidence of unintentional overdose of the product and the rapid regression of symptoms after its withdrawal. Owing to the fact that there are only a few similar cases described in the literature, we assume that such episodes will rarely occur in clinical practice. Apparently, while we do not have enough data, it is impossible to predict the appearance of the described symptoms in patients who use minoxidil solutions. Despite that, it is important to take into account the chance of cardiovascular symptoms following minoxidil use.

To the best of our knowledge, the mechanism of systemic absorption of minoxidil during topical use, the factors affecting the level of absorption, and the mechanism for the development of systemic side effects are not reliably known. In addition, it is not known for certain what blood concentration of minoxidil is sufficient for developing cardiovascular side effects. To obtain a complete understanding of the causes and frequency of systemic side effects of topical minoxidil, further study of the problem is necessary.

## Conclusions

In most cases of any cardiovascular disorder associated with the use of topical minoxidil, it is complicated to prove the relationship between the use of minoxidil and the resulting disorders. This case illustrates the potential for systemic cardiovascular effects resulting from topical minoxidil use, albeit in large amounts. The possible occurrence of undesirable effects on the cardiovascular system should be considered when prescribing topical products containing minoxidil. To form a reliable understanding of the causes and frequency of systemic effects when topical minoxidil is used, further investigation of the problem is required.

## References

[1] Nestor MS, Ablon G, Gade A, Han H, Fischer DL (2021). Treatment options for androgenetic alopecia: efficacy, side effects, compliance, financial considerations, and ethics. J Cosmet Dermatol.

[2] Dubrey SW, VanGriethuysen J, Edwards CM (2015). A hairy fall: syncope resulting from topical application of minoxidil. BMJ Case Rep.

[3] Korbi M, Said El Mabrouk R, Abdelaali M, Youssef M, Belhadjali H, Zili J (2022). Topical minoxidil-induced paresthesia. Dermatol Ther.

[4] Venkatesh R, Pereira A, Jain K, Yadav NK (2020). Minoxidil induced central serous Chorioretinopathy treated with oral eplerenone - a case report. BMC Ophthalmol.

[5] Linas SL, Nies AS (1981). Minoxidil. Ann Intern Med.

[6] Rossi A, Cantisani C, Melis L, Iorio A, Scali E, Calvieri S (2012). Minoxidil use in dermatology, side effects and recent patents. Recent Pat Inflamm Allergy Drug Discov.

[7] Ranchoff RE, Bergfeld WF (1985). Topical minoxidil reduces blood pressure. J Am Acad Dermatol.

[8] Bokhari L, Jones LN, Sinclair RD (2022). Sublingual minoxidil for the treatment of male and female pattern hair loss: a randomized, double-blind, placebo-controlled, phase 1B clinical trial. J Eur Acad Dermatol Venereol.

[9] Vahabi-Amlashi S, Layegh P, Kiafar B (2021). A randomized clinical trial on therapeutic effects of 0.25 mg oral minoxidil tablets on treatment of female pattern hair loss. Dermatol Ther.

[10] Asilian A, Farmani A, Saber M (2024). Clinical efficacy and safety of low-dose oral minoxidil versus topical solution in the improvement of androgenetic alopecia: a randomized controlled trial. J Cosmet Dermatol.

[11] Beach RA, McDonald KA, Barrett BM, Abdel-Qadir H (2021). Side effects of low-dose oral minoxidil for treating alopecia. J Am Acad Dermatol.

[12] Gheshlaghi F, Zoofaghari S, Dorooshi G (2018). Unstable angina: a rare presentation of minoxidil intoxication: a case report and literature review. J Res Pharm Pract.

[13] Oye M, Oye M, Ali A (2021). Signs of early cardiac tamponade induced by Minoxidil. Am J Emerg Med.

[14] Shen Y, Zhu Y, Zhang L, Sun J, Xie B, Zhang H, Song X (2023). New target for minoxidil in the treatment of androgenetic alopecia. Drug Des Devel Ther.

